# Obeticholic acid prevents cyclophosphamide-induced placental injury via SIRT1 and TLR4/NF-κB pathways

**DOI:** 10.1186/s40360-025-00986-0

**Published:** 2025-09-19

**Authors:** Walaa Yehia Abdelzaher, Hanaa Mohamed Khalaf, Sara M. Ahmed, Mina Ezzat Attya, Abdelaleem Abdelnour Mohamed, Asmaa Mohamed Mahmoud Ali, Shereen S. Gaber, Ahmed K. A. Abdel-Hakeem, Enas Mostafa Mohammed, Marwa Hassan

**Affiliations:** 1 Department of Medical Pharmacology, Faculty of Medicine, Minia University, Minia, 61519 Egypt; 2 Faculty of Nursing, Lotus University, 61768 Minia, Egypt; 3 Department of Pathology, Faculty of Medicine, Minia University, Minia, 61519 Egypt; 4 Department of Medical Physiology, Faculty of Medicine, Minia University, Minia, 61519 Egypt; 5 Department of Forensic Medicine and Clinical Toxicology, Faculty of Medicine, Minia University, Minia, 61519 Egypt; 6 Department of Medical Biochemistry, Faculty of Medicine, Minia University, Minia, 61519 Egypt; 7 Department of Obstetrics & Gynecology, Faculty of Medicine, Minia University, Minia, 61519 Egypt

**Keywords:** Obeticholic acid, Cyclophosphamide, Placenta, SIRT1, TLR4

## Abstract

**Background:**

The aim of the current study is to identify the possible protective effect of obeticholic acid (OCA) on placental injury caused by cyclophosphamide (CP). OCA was administered in the presence and absence of CP.

**Methods:**

Thirty-two pregnant female rats were randomly assigned to four groups: control group, OCA group: received OCA (10 mg/kg /day, orally), CP group: received CP 20 mg/kg intraperitoneally at 12th day, OCA + CP group. Placental weight and placental growth factor (PlGF) were measured. Placental oxidative stress parameters (malondialdahide (MDA) and total antioxidant capacity (TAC)), besides Sirtuin type 1 (SIRT1), nuclear factor erythroid 2-related factor 2 (Nrf2), myeloid differentiation factor 88 (Myd88) and caspase-3 biomarkers, were evaluated. Nuclear factor-kappa B (NF-κB) and tumor necrosis factor-alpha (TNF-α) gene expression were also measured. Placental histopathological examination, toll- like receptor4 (TLR4) and forkhead-box transcription factor1 (FOXO1) immunohistochemical study were performed.

**Results:**

CP significantly decreased PlGF, placental weight, TAC, SIRT1 and Nrf2 with increased placental MDA, Myd88, caspase-3, NF-κB and TNF-α. Histopathological findings of placental damage and high TLR4, FOXO1 immunoexpressions were detected. OCA significantly increased PlGF level, placental weight and normalized the distributed oxidative stress, inflammatory, and apoptotic biomarkers with a prompt improvement in the histopathological picture and decrease TLR4 and FOXO1 immunoexpressions.

**Conclusion:**

Accordingly, these findings suggest that OCA protects CP-induced placental injury by modulating TLR4/Myd88/NF-κB; SIRT1-dependant signaling pathways.

**Graphic abstract:**

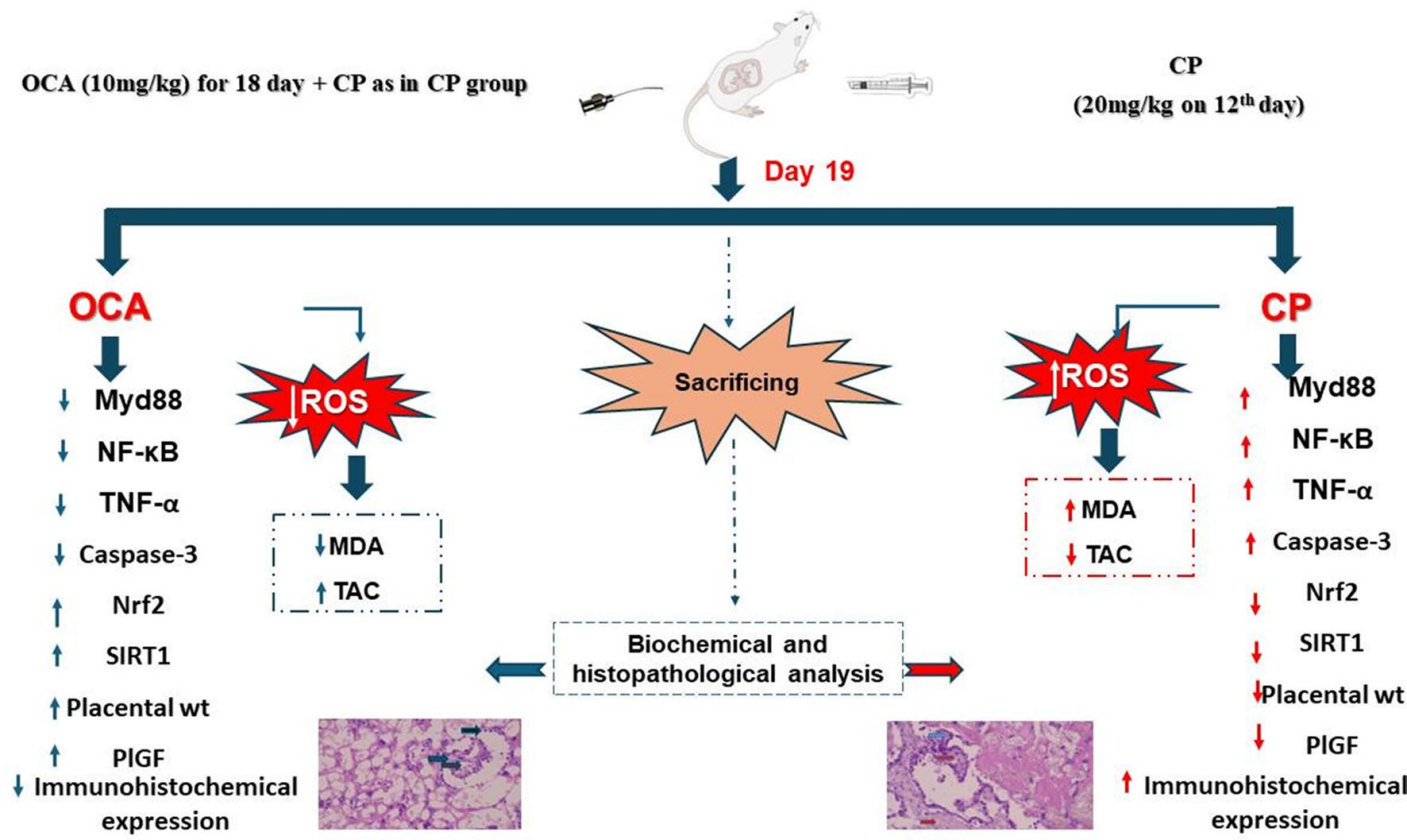

## Background

The placenta serves as the fetus’s respiratory, digestive, and excretory organs throughout pregnancy. It also performs endocrine and immunological tasks that are essential for a healthy pregnancy. It is a transient organ that is crucial to healthy embryonic development. It serves as the connection between the mother and her fetus [1]. Placental dysfunction and injury have an impact on fetal growth and pregnancy continuity. An important hint for assessing the mechanisms of prenatal toxicity is the chemical induction of placental damage [2].

Chemotherapy medications are commonly used to treat autoimmune diseases and cancer, but they have a double-edged effect since they are non-selective, affecting both healthy cells and tumors, which can lead to systemic damage. Cyclophosphamide (CP) is one of these chemotherapy drugs. CP is an effective medication used to treat multiple sclerosis, systemic lupus erythematosus (SLE), and a variety of malignancies. It also functions as an immunosuppressive agent in organ transplantation. CP is mostly used to treat SLE in pregnant women; however it still has teratogenic effects despite the possibility of successful pregnancies following exposure [3, 4].

CP has numerous harmful effects on healthy cells in both humans and lab animals, despite its many advantages. CP has an adverse effect on oogenesis and spermatogenesis. Additionally, it causes fetal and placental damage during pregnancy by crossing the placenta. Shortly after injection, CP promotes apoptosis in embryonic neural progenitor cells of the telencephalon by stopping the S-phase of the cell cycle [5].

The placenta expresses Sirtuin type 1 (SIRT1), a histone deacetylase that is dependent on nicotinamide adenine dinucleotide þ (NADþ). It can control the majority of bodily physiological functions, interacts with protein substrates to play a crucial role in several signaling pathways, and is required for metabolism, apoptosis, cell division, and proliferation. Numerous studies have demonstrated that SIRT1 plays a part in the toxic harm that many medications, including CP, cause to various organs. Because of its anti-inflammatory and antioxidant properties, nuclear factor (erythroid-derived 2)-like 2 (Nrf2) has a cytoprotective effect. SIRT1 by engaging with other protein substrates and the family of Forkhead-box transcription factors (FOXO), the placenta, testes, ovary, prostate, lung, and pancreas are among the organs that express the FOXOs protein family, which includes FOXO1, FOXO3, FOXO4, and FOXO6. The regulation of cellular metabolic pathways that impact the cell cycle, oxidative stress response, and cell death depends on these proteins. Depending on blood glucose levels, FOXO1 regulates angiogenesis, cell survival, or proliferation during placenta development [6]. The control of the SIRT1/FOXO1; SIRT1/Nrf2/ tumor necrosis factor-α (TNF-α) pathways may be essential for the treatment of rat placental injury.

On the other hand, Toll-like receptors (TLR), which include 10 members, are the most well-known transmembrane recognition proteins in humans. Among these is Toll-like receptor 4 (TLR4), which is essential for the inflammatory response observed in experimental placental injury. TLR4 stimulation causes myeloid differentiation factor 88 (Myd88) to attract inflammatory cells, which in turn triggers the release of proinflammatory mediators such as interleukin-1β (IL-1β), TNF-α and nuclear factor-kappa B (NF-κB) [7]. Modification of the TLR4/Myd88/NF-κB signaling pathway may explain mechanisms of placental damage and protection.

SIRT1 and TLR4 were likely chosen in CP-induced placental injury because they represent upstream, mechanistically relevant pathways; involved in placental health, inflammation, and oxidative stress and offer potential therapeutic targets and biomarkers of CP-induced toxicity [8, 9].

Obeticholic acid (OCA), a semi-synthetic bile acid analogue and farnesoid X receptor (FXR) agonist, is the most clinically advanced of those derivatives. It reduces inflammation and hepatic steatosis by preventing the activation of the NLRP3 inflammasome in the liver and macrophages [10]. OCA therapy reduces inflammation and hepatic steatosis in the nonalcoholic steatohepatitis model [11]. According to Fiorucci et al. [12], activation of OCA also has a number of biological consequences, such as decreasing lipogenesis, suppressing the production of de novo cholesterol, and increasing the β-oxidation of long-chain FAs. OCA has been demonstrated to have anti-inflammatory and antioxidant properties [13]. OCA has also been shown to have anti-diabetic effects [14], cardiovascular protection [10, 11].

With regard to OCA’s pleiotropic qualities, the current study investigated its ability to treat CP-induced placental toxicity via modulating the SIRT1/Nrf2/TNF-α; SIRT1/FOXO1 and TLR4/Myd88 /NF-κB signaling pathways.

## Methods

### Ethics

The NIH Guide for the Care and Use of Laboratory Animals and the Institutional Ethical Committee’s (Faculty of Medicine, Minia University, Egypt) recommendations for the care of experimental animals were followed when handling, administering medication, and authenticating the animals. (Approval number: 1342/11/2024).

### Chemicals

OCA was purchased from (Marcyrl Pharmaceutical Industries, Egypt) and CP was obtained from (Zydus Cadila Co., India). The remaining compounds were all obtained from commercial sources and belonged to the highest ana lytical grade.

## Animals and experimental design (Fig. 1)

**Fig. 1:**
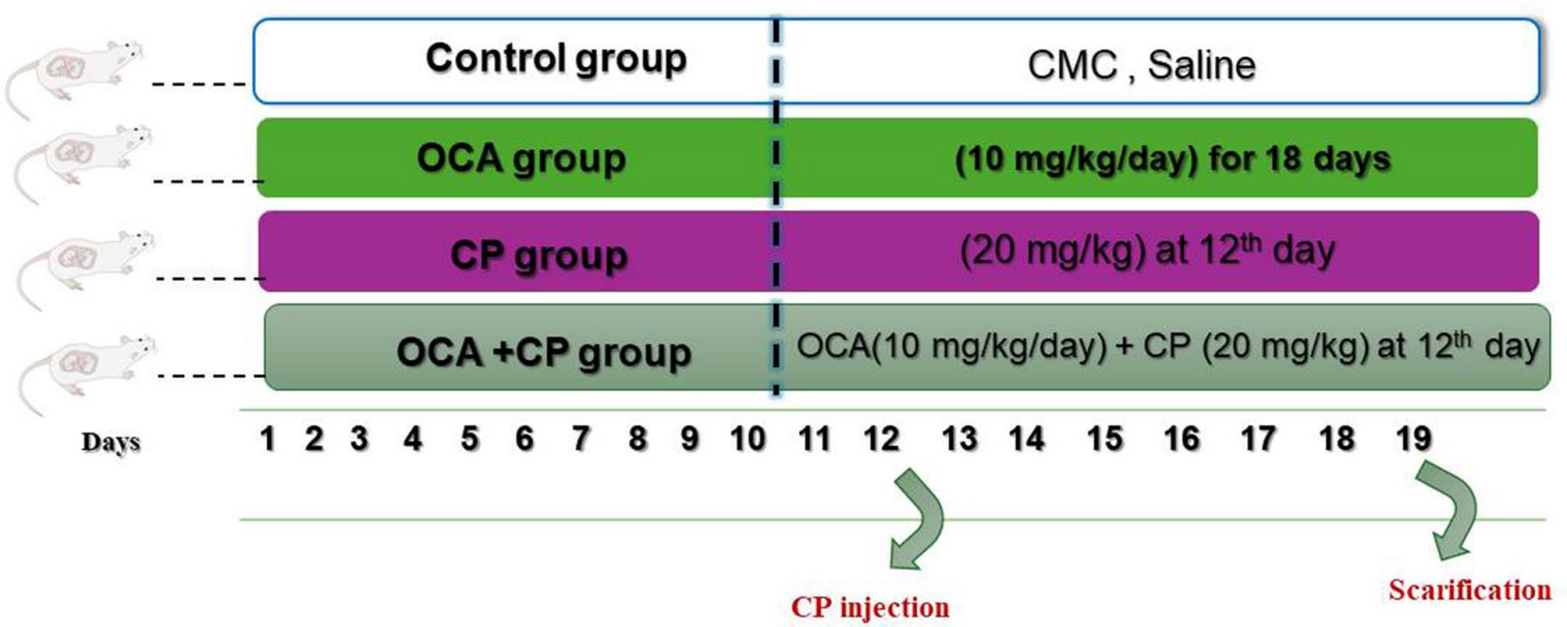
Scheme of experimental design

### Animals

The National Research Centre in Giza, Egypt, provided 36 adult female Sprague Dawley rats (12–15 weeks old, 200–250 g) and 18 adult male Sprague Dawley rats (12–14 weeks old, 200–250 g). The rats were kept in the department of Medical Pharmacology’s animal home at Minia University in Egypt’s Faculty of Medicine.

### Mating procedure and experimental groups

Overnight, male rats mated with all female rats. The day the spermatozoa were found in the vaginal smear was identified as day 0 of pregnancy. Before a duplicate number of groups was achieved, the mating time lasted for 15 days in a row. Nevertheless, female rats that were not mated during this time were deemed infertile and removed from the study [15]. With a 90% success rate in mating, there were thirty two pregnant female rats in total. Rats were then randomly assigned to four groups, with eight rats in each:

#### Control pregnant group

Pregnant rats were given 1 milliliter of carboxymethylcellulose (CMC), a vehicle of OCA from El-Nasr Pharmaceutical Co., Egypt, for 18 days of gestation. On the 12th day of gestation, the rats received an intraperitoneal (i.p.) saline injection, which is the solvent of CP.

#### Obeticholic acid pregnant group (OCA group)

Pregnant rats received OCA (10 mg/kg /day, orally) [11, 16] suspended in 1 ml CMC for 18 days of gestation.

#### Cyclophosphamide pregnant group (CP group)

On day 12th of pregnancy, pregnant rats were given a single intraperitoneal (20 mg/kg) dosage of CP dissolved in saline [2, 17].

#### Obeticholic acid -cyclophosphamide pregnant group (OCA + CP group)

For 18 days of gestation, pregnant rats were given OCA suspended in 1 ml CMC (10 mg/kg orally/day), and on the 12th day of gestation, they were given a single dosage of CP (20 mg/kg, i.p.).

### Sample collection

On day 19 of gestation, all rats were euthanized under light halothane anesthesia. To access the gravid uterine horns, an incision was made in the lower midline abdominal cavity. Each horn had an average of three sacs (placentae in a rat with six live pups average six). Fetal membranes were cut away from the placenta’s attachment site, all the sacs were opened, the fetus and umbilical cord were visible, and the placentae were separated and weighed. Placentae were separated into two sections: the first was preserved in 10% formal saline for 48 h in order to perform histopathological and immunohistochemical analyses; the second was homogenized in a 10 mM phosphate buffer solution (pH 7.4), where the placenta weight to buffer solution volume ratio was 1:5. The supernatant was then centrifuged for 15 min at 4000 rpm and used for placental biochemical analysis.

## Biochemical determination

### Estimation of lung oxidant/antioxidant, inflammatory, and apoptotic parameters

A colorimetric commercial kit was used to assess malondialdehyde (MDA) and total antioxidant capacity (TAC) in accordance with the guidelines provided by Bio Diagnostic Co., Egypt (Catalog No: MD2529 and TA2513, respectively).

SIRT1, Nrf2, and caspase-3 were measured using ELISA kits from MyBioSource, San Diego, California, USA (Catalog No: MBS2600246, MBS752046, and MBS261814, respectively). Myd88 level was also evaluated by ELISA kits (ELISA Genie Co., Dublin, Ireland, Catalog No: SKU: RTFI01303).

### Assessment of placental growth factor

Rat PlGF ELISA kit (Sandwich ELISA kit, Assay Genieco, Ireland) was used to quantify placental growth factor (PlGF) in accordance with the manufacturer’s instructions (Catalog No: RTDL00847).

### Real-time reverse-transcription polymerase chain reaction (RT-PCR) of placental TNF-α and NF-κB gene expression

One milliliter of TRIzol reagent was used to ultrasonically homogenize 100 milligrams of placental tissue using a Branson Digital Sonifer ultrasonic cell homogenizer (SFX 550, Danbury, Connecticut, United States). Samples with purity ≥ 1.7 based on the ratio of A260/A280 were used in this work using the Revert Aid First Strand cDNA Synthesis Kit (Catalog No: K1622, Thermofisher Scientific, United Kingdom) [18]. For every sample, a same amount of total RNA was used to create cDNA. For qRT-PCR, single-stranded cDNAs were employed. The primer sets that were utilized were TNF-α forward, 5′-CCTCTCTGCCATCAAGAGCC-3′, and reverse, 5′-GGCTGGGTAGAGAACGGATG-3′. NF-κB forward, 5′-CAGCAGATGGCCCATACCTT-3′, and reverse, 5′-GTTT GCAAAGCCAACCACCA-3′. β-actin forward primers are as follows: 5′-GACGAGGCCCAGAGCAAGAGAGG-3′ and reverse 5′-GAT CCA CAT CTG CTG GAA GGT GGA C-3′. The Step One real-time PCR Detection System (Ref no: 4369074, Applied Biosystems, Singapore) was used to perform qRT-PCR and Thermo Scientific Maxima SYBR Green qPCR Master Mix (2X) (Catalog No: K0251, Thermofisher Scientific, United Kingdom). The relative gene expression was measured using the housekeeping gene, β-actin, and the mRNA levels were analyzed using a comparable cycle threshold technique.

### Histopathology and immunohistochemical study

#### Histological procedures

After a day in a 10% formalin solution, the placentas of the pregnant female rats were processed and embedded in paraffin to create paraffin blocks. A 5 μm thick cross section was cut with a microtome. These tissue sections were stained with hematoxylin-eosin stain and subsequently histopathologically analyzed using an Olympus light microscope blindly.

### Immunohistochemistry

Each placenta was sliced into 5 μm slices, and tissue blocks embedded in formalin-fixed paraffin were put on positively charged glass slides for manual immunohistochemistry labeling. Tissue sections were first rehydrated and deparaffinized. The slides were then submerged in a 3% hydrogen peroxide solution for 30 min in order to suppress the endogenous peroxidase. The slides were submerged in sodium citrate buffer (pH 6.0) for 10 min each for two passes in a 700-W microwave to retrieve the antigen. The slides were coated with an ultraviolet block to prevent the non-specific background staining. After adding the anti-TLR 4 and anti-FOXO 1 polycolonal rabbit antibodies (ABclonal Laboratories, China) to the slides in 1:100 dilutions, they were incubated for 24 h at 4 °C in a humidity chamber. Tissue sections on the slides were treated with the secondary antibody for 30 min. Each slide was coated with DAB substrate-chromagen, and tissue sections were then counter-stained with Mayer’s haematoxylin.

#### Scoring of immunohistochemical staining

Regarding evaluation and scoring of both TLR 4 and FOXO 1 immunohistochemical expressions, the slides were examined under light microscope magnification x200 or x400. TLR 4 immuno-expression was scored into score (0) for absent expression; score (1) for weak intensity expression score (2) for moderate intensity expression score (3) for strong intensity expression [19]. Foxo 1 immuno-expression was scored according to staining intensity into score (0) for absent stain; score (1) for weak intensity expression score (2) for moderate intensity expression score (3) for strong intensity expression [20].

### Statistical analysis

Following a one-way ANOVA, Tukey’s multiple comparison tests were used. The results were displayed as means ± SEM. For analysis, GraphPad Prism version 5 was used. P-values were deemed significant if they were less than 0.05.

## Results

### Effect of OCA on placental weight (wt) and PlGF in CP-induced placental toxicity

**Fig. 2:**
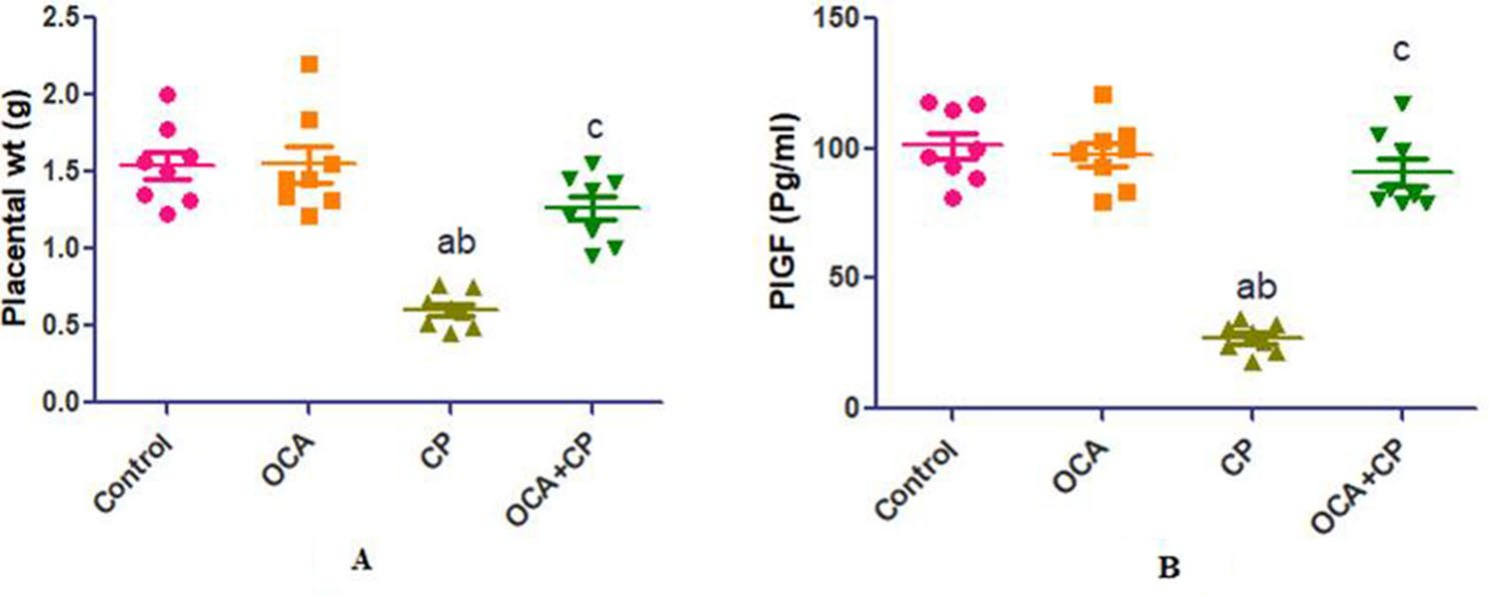
Effect of OCA on placental wt and PlGF in CP-induced placental toxicity. Data are expressed as mean ± SEM. (*n* = 8 rats/group (.Values were considered significantly different when *P* < 0.05. ^a^Significant difference from control group, ^b^significant difference from OCA group, ^c^significant difference from CP group. (OCA = obeticholic acid; CP = cyclophosphamide; PlGF = placental growth factor)

Figure 2 demonstrated that placental wt and PlGF showed significant decrease in CP group when compared to control and OCA groups. On the other hand, OCA + CP group showed significant increase in both when compared to CP group.

### Effect of OCA on placental oxidative stress parameters in CP-induced placental toxicity

CP group showed a significant increase in placental MDA with a significant decrease in placental TAC when compared to control and OCA groups. In contrast to the CP group, the OCA + CP group demonstrated a notable improvement in oxidative stress parameters (Table 1).

**Table 1:** Effect of OCA on placental oxidative stress parameters in CP-induced placental toxicity

Group	Placental TAC (mmol/L)	Placental MDA (nmol/g tissue)
Control	45.62 ± 2.06	34.75 ± 2.28
OCA	46.75 ± 2.51	37.54 ± 2.47
CP	20.70 ± 1.19^ab^	97.69 ± 3.25 ^ab^
OCA + CP	41.14 ± 3.18^c^	42.85 ± 1.81 ^c^

Data are expressed as mean ± SEM. (*n* = 8 rats/group (.Values were considered significantly different when *P* < 0.05. ^a^Significant difference from control group, ^b^significant difference from OCA group, ^c^significant difference from CP group. (OCA = obeticholic acid; CP = cyclophosphamide; TAC = total antioxidant capacity; MDA = malondialdehyde)

### Effect of OCA on placental anti-inflammatory and anti-apoptotic parameters in CP-induced placental toxicity

**Fig. 3:**
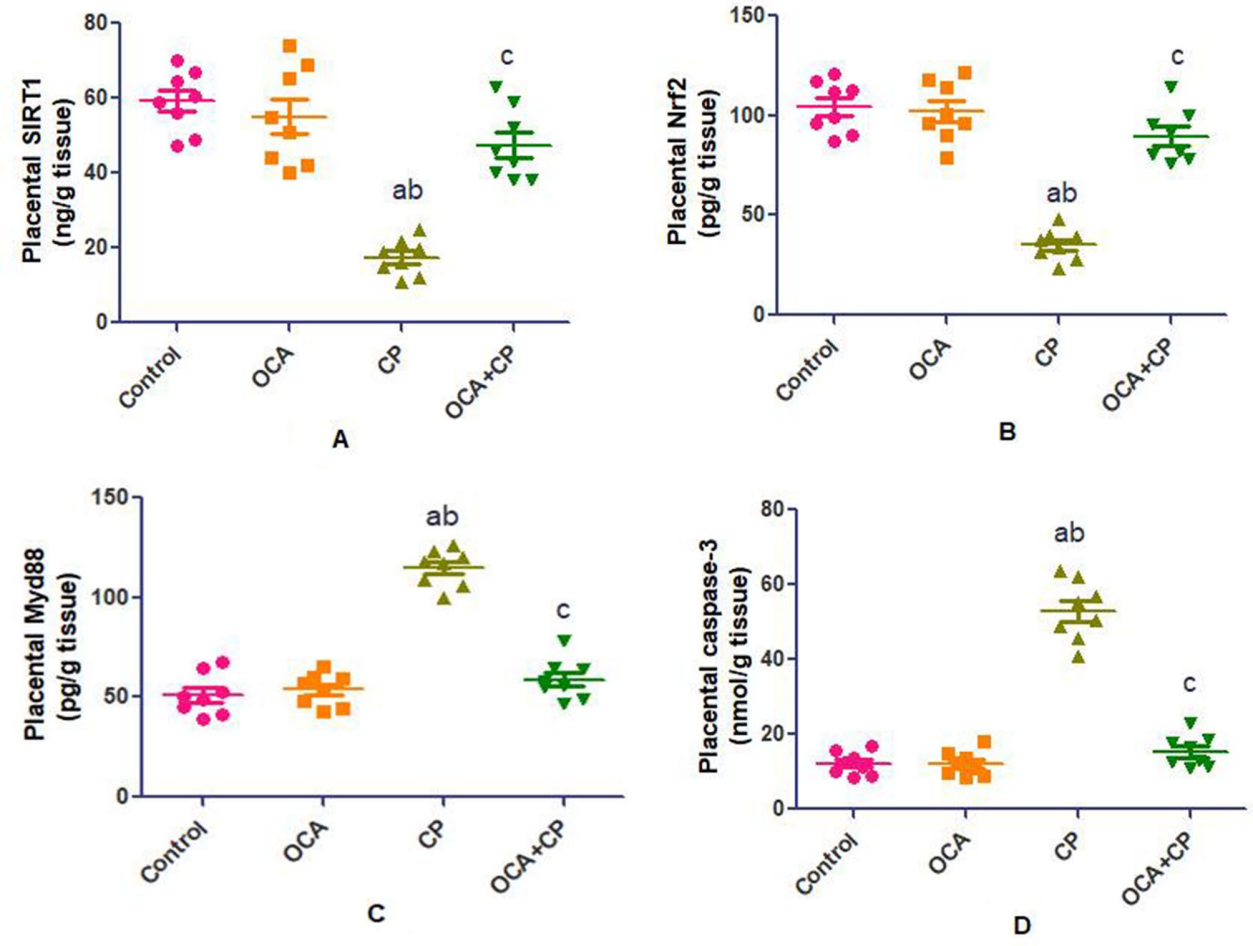
Effect of OCA on placental anti-inflammatory and anti-apoptotic parameters in CP-induced placental toxicity. Data are expressed as mean ± SEM. (*n* = 8 rats/group (.Values were considered significantly different when *P* < 0.05. ^a^Significant difference from control group, ^b^significant difference from OCA group, ^c^significant difference from CP group. (OCA = obeticholic acid; CP = cyclophosphamide; SIRT1 = Sirtuin type 1; Nrf2: nuclear factor erythroid 2-related factor 2; Myd88 = myeloid differentiation factor 88)

**Fig. 4:**
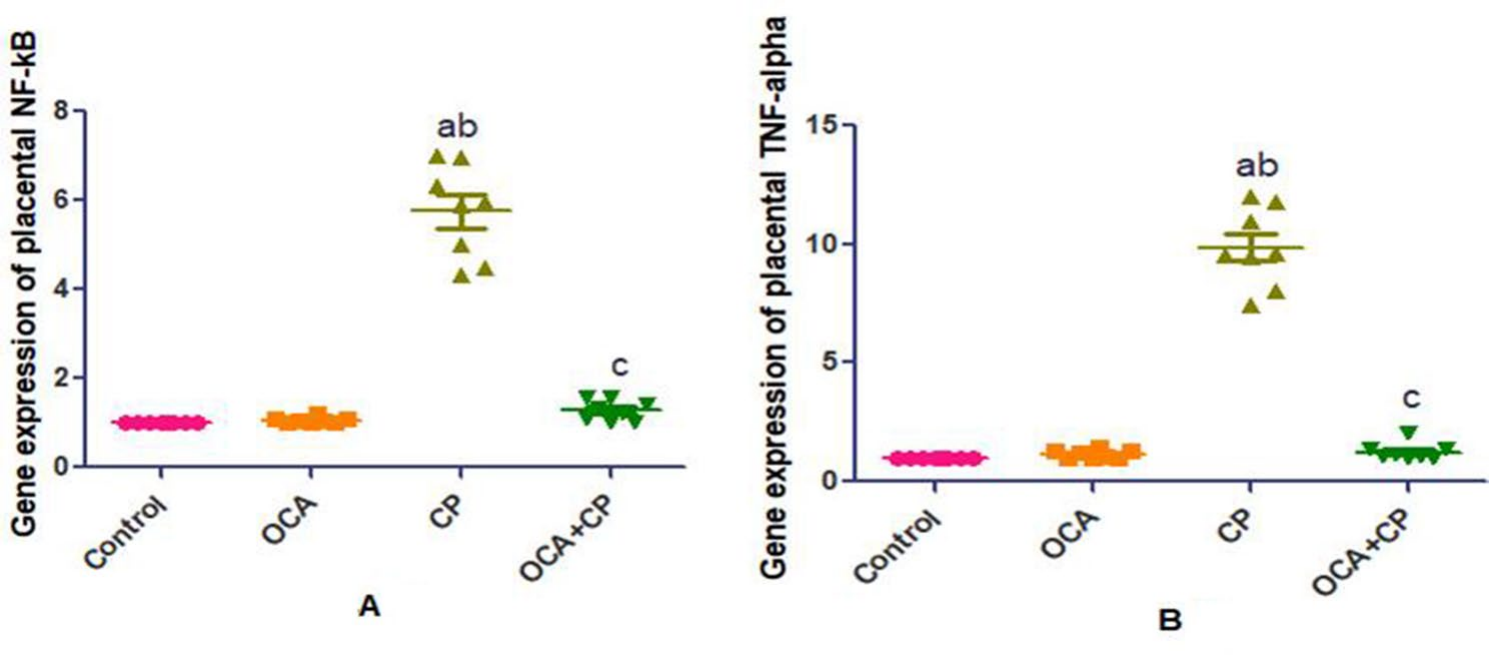
Effect of OCA on placental NF-κB and TNF-α gene expression in CP-induced placental toxicity. Data are expressed as mean ± SEM. (*n* = 8 rats/group (.Values were considered significantly different when *P* < 0.05. ^a^Significant difference from control group, ^b^significant difference from OCA group, ^c^significant difference from CP group. (OCA = obeticholic acid; CP = cyclophosphamide; NF-κB = nuclear factor-kappa B; TNF-α = tumor necrosis factor-alpha)

Placental SIRT1 and Nrf2 showed significant decrease with significant increase in placental Myd88 and caspase-3 levels (Fig. 3); NF-κB and TNF-α gene expression (Fig. 4) in CP group when compared to control and OCA groups. As compared to CP group, the OCA + CP group showed significant improvement in placental anti-inflammatory and anti-apoptotic parameters. The balance between these measurements significantly dictates the extent of placental injury and recovery.

### Histological results

**Fig. 5:**
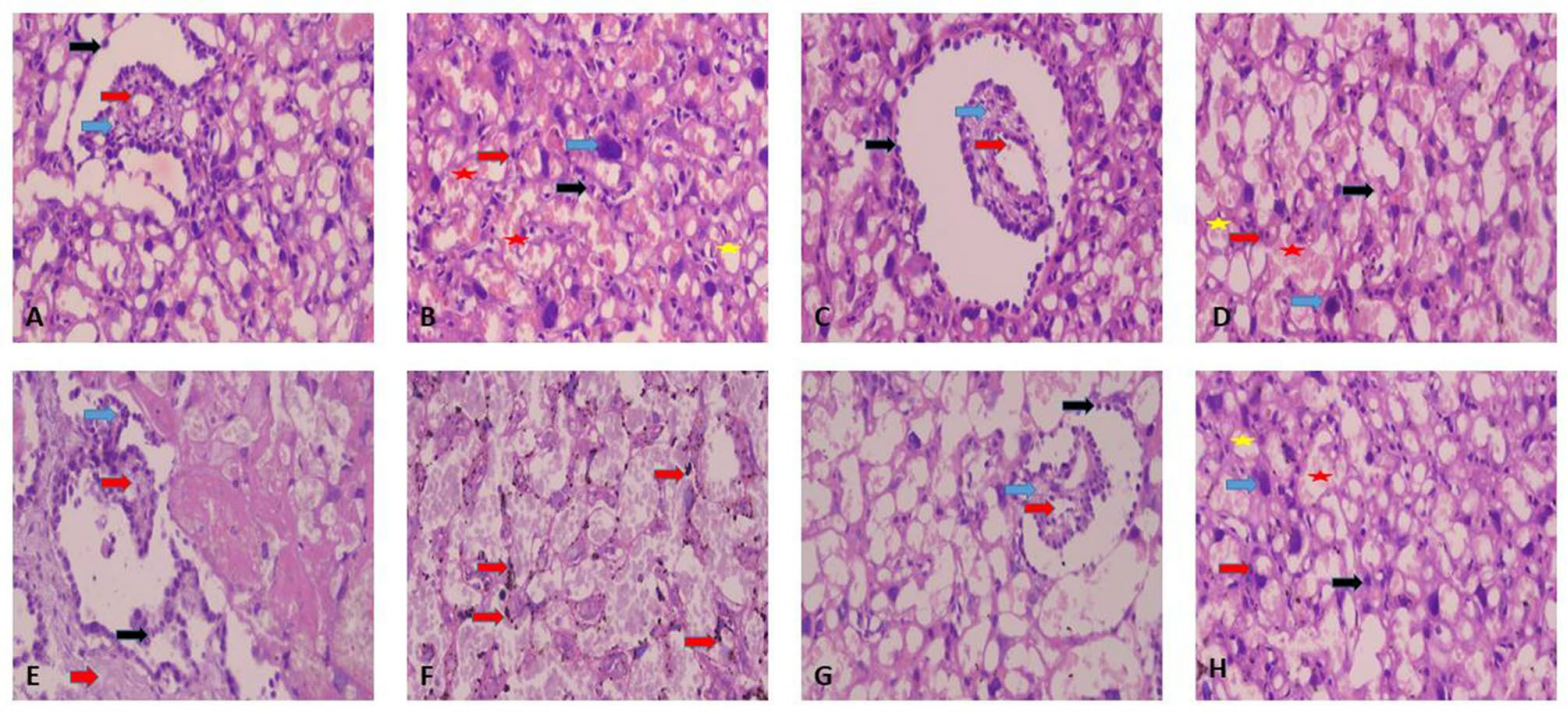
Effect of OCA on placental histopathology in CP-induced placental toxicity. Both figures (**A**) and (**B**) represent control group. Figure (**A**) shows normal chorionic villi composed of external trophoblastic tissue (black arrow), intermediate connective tissue (blue arrow) and internal fetal blood vessels (red arrow. Figure (**B**) shows normal labyrinth zone of the placenta with normal both maternal large sized sinuses (red star) and fetal small sized capillaries (yellow star) separated from each other’s by intact interhaemal membrane (red arrow) with normal both cytotrophoblasts (blue arrow) and syncytiotrophoblasts (black arrow). Both figures (**C**) and (**D**) represent drug OCA group. Figure (**C**) shows normal chorionic villi composed of external trophoblastic tissue (black arrow), intermediate connective tissue (blue arrow) and internal fetal blood vessels (red arrow). Figure (**D**) shows normal labyrinth zone of the placenta with normal both maternal sinuses (red star) and fetal capillaries (yellow star) separated from each other’s by intact interhaemal membrane (red arrow) with normal both cytotrophoblasts (blue arrow) and syncytiotrophoblasts (black arrow). Both figures (**E**) and (**F**) represent CP group. Figure (**E**) shows injured placental tissue distorted chorionic villi (blue arrow) with syncytial knots formation (black arrow) and edematous mesenchymal connective tissue (red arrow). Figure (**F**) shows with labyrinth zone with diffuse intra trophoblastic haemosidern deposition (red arrow). Both figures (**G**) and (**H**) represent OCA + CP group. Figure (**G**) shows chorionic villi composed of normal external trophoblastic tissue (black arrow), and internal fetal blood vessels (red arrow) with minimal edema of the connective tissue (blue arrow). Figure (**H**) shows normal labyrinth zone of the placenta with normal both maternal large sized sinuses (red star)and fetal small sized capillaries (yellow star) separated from each other’s by intact interhaemal membrane (red arrow) with normal both cytotrophoblasts (blue arrow) and syncytiotrophoblasts (black arrow) with few foci of hemosiderin deposition

Sections examined from both control and OCA groups showed normal placental tissue composed of normal chorionic villi and labyrinth zone. The normal chorionic villi were composed of external trophoblastic tissue, intermediate connective tissue and internal fetal blood vessels. The normal labyrinth zone of the placenta were formed of normal both maternal large sized sinuses and fetal small sized capillaries separated from each other by intact interhaemal membrane with normal both cytotrophoblasts and syncytiotrophoblasts. Sections examined from CP group showed injured placental tissue in the form of distorted chorionic villi with syncytial knots formation and edematous mesenchymal connective tissue. The labyrinth zone showed diffuse intra trophoblastic haemosidern deposition. Sections examined from OCA + CP group showed improvement of the placental injury. The chorionic villi were composed of normal external trophoblastic tissue and internal fetal blood vessels with minimal edema of the connective tissue. The labyrinth zone were formed of normal both maternal large sized sinuses and fetal small sized capillaries separated from each other by intact interhaemal membrane with normal both cytotrophoblasts and syncytiotrophoblasts with few foci of hemosiderin deposition (Fig. 5).

**Fig. 6:**
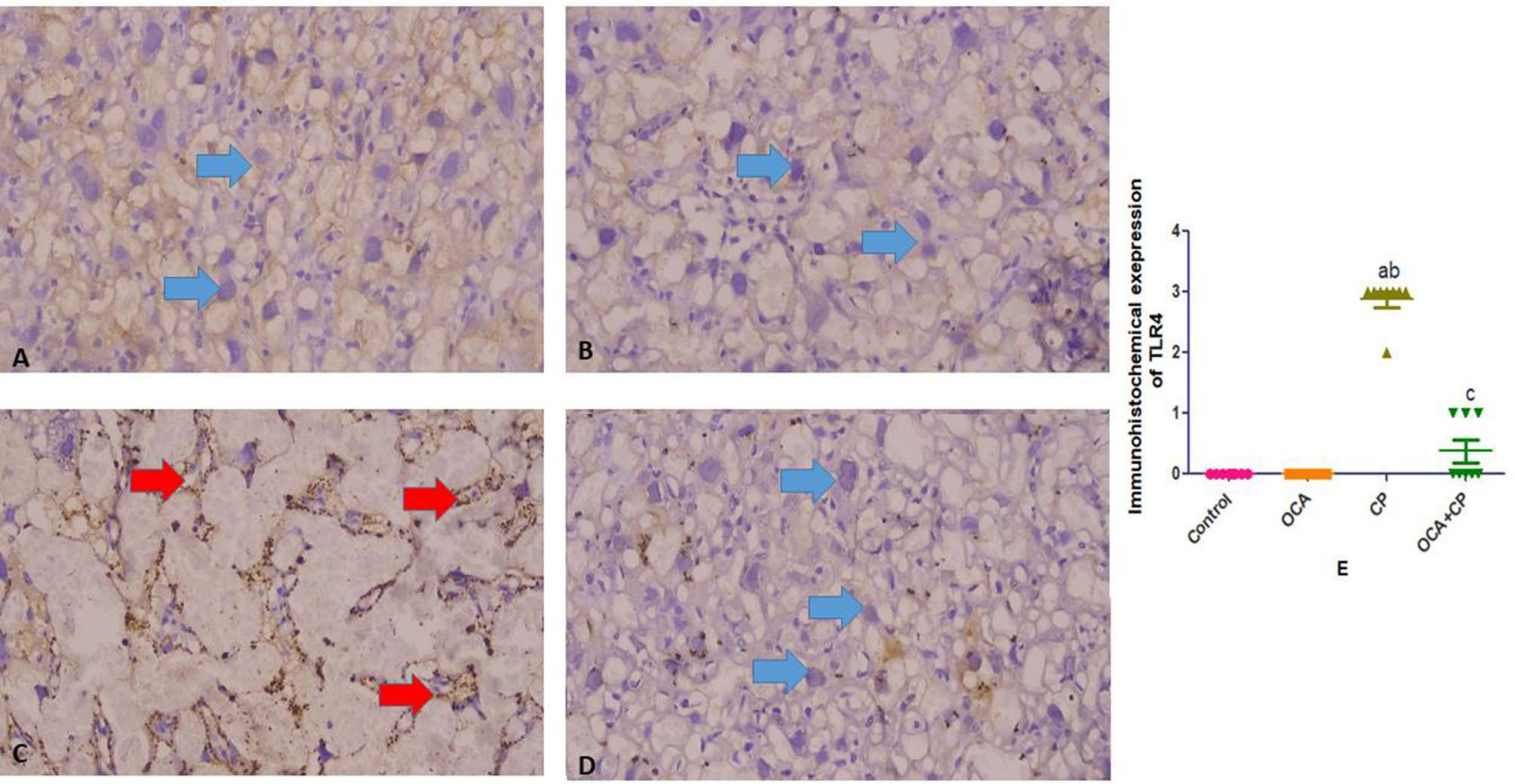
Immunohistochemistry expression of TLR4. Figure (**A**): negative TLR 4 expression in control group (blue arrow). Figure (**B**): negative TLR 4 expression in OCA group (blue arrow). Figure (**C**): positive TLR 4 expression in CP group (red arrow). Figure (**D**): negative TLR 4 expression in OCA + CP group with reduced the immunoreactivity than CP group (blue arrow). Figure (**E**): Immunohistochemical expression scoring; Data represent the mean ± SEM. P-value < 0.05 is set for significance

**Fig. 7:**
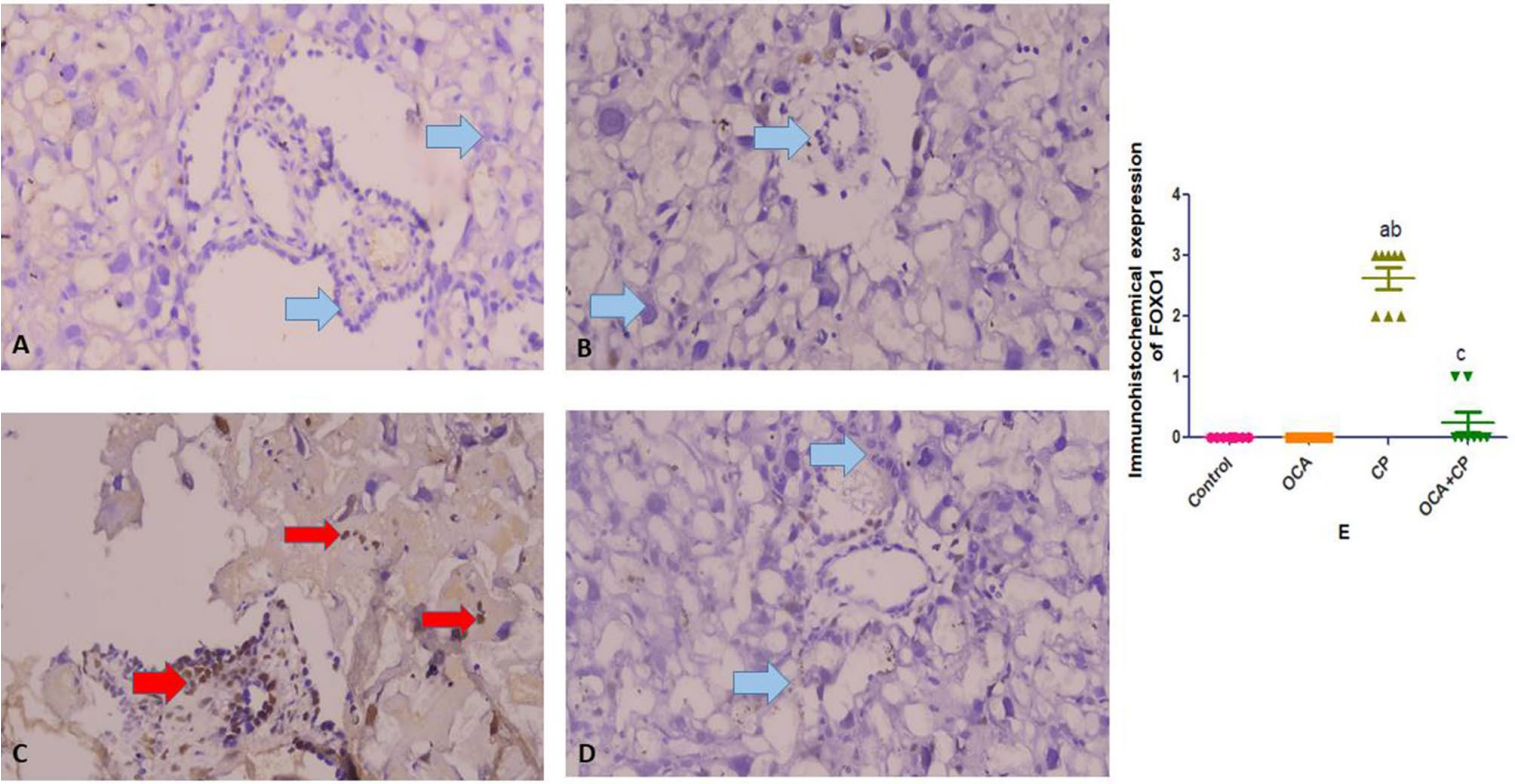
Immunohistochemistry expression of FOXO1. Figure (**A**): negative Foxo 1 expression in control group (blue arrow). Figure (**B**): negative Foxo 1 expression in OCA group (blue arrow). Figure (**C**): positive Foxo 1 expression in CP group (red arrow). Figure (**D**): negative Foxo 1 expression in OCA + CP group with reduced the immunoreactivity than CP group (blue arrow). Figure (**E**): Immunohistochemical expression scoring; Data represent the mean ± SEM. P-value < 0.05 is set for significance

Both control and OCA groups showed negative expressions of both TLR 4 and FOXO 1. CP group showed strong expressions of both TLR 4 and FOXO 1. While treated OCA + CP group showed reduced immunoreactivity of cells to both TLR 4 and FOXO 1 expressions. Immunohistochemical expression scoring; Data represent the mean ± SEM. P-value < 0.05 is set for significance (Figs. 6 and 7; respectively).

## Discussion (Fig. 8)

**Fig. 8:**
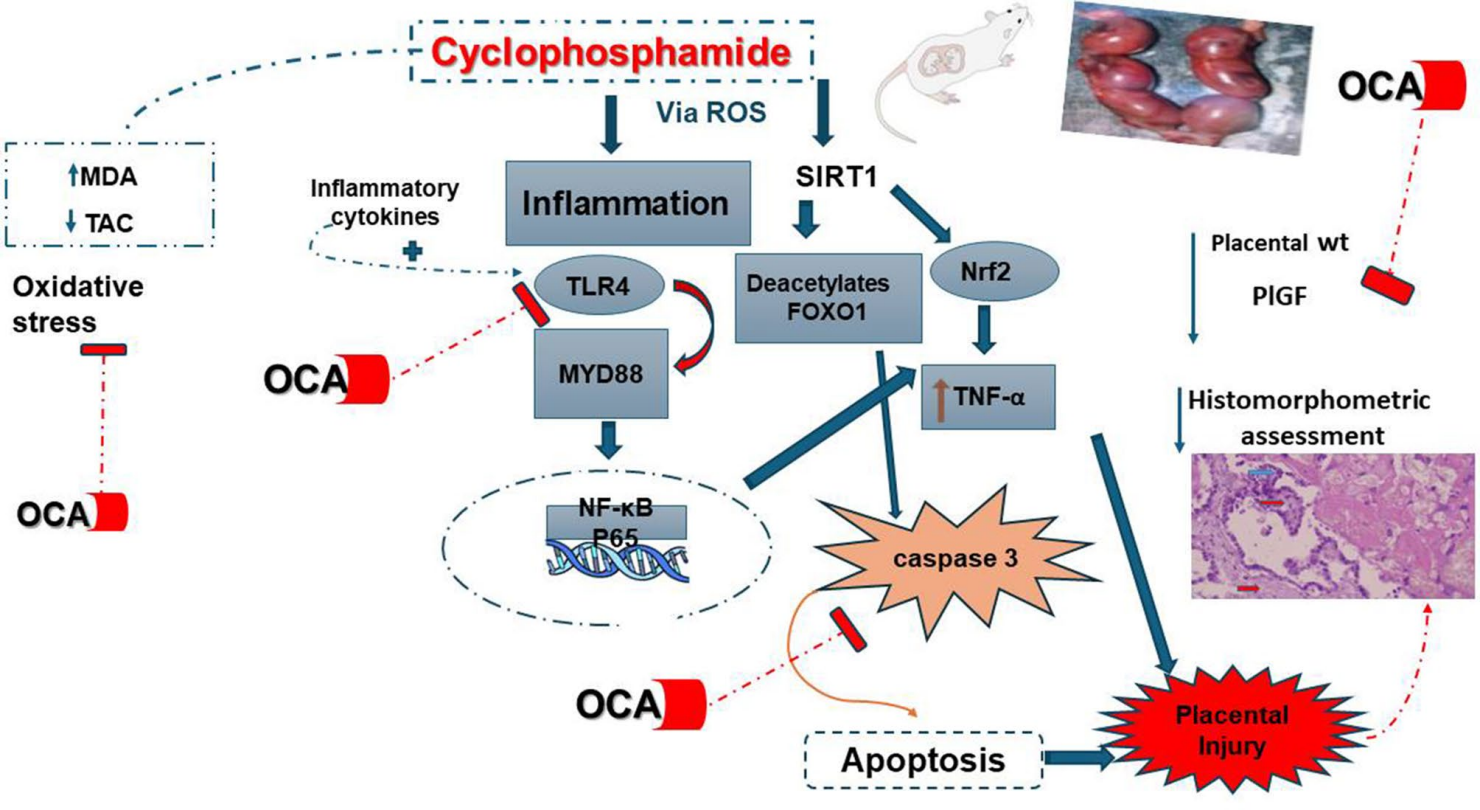
Graphical summary mapping how OCA modulates the interlinked signaling pathways. OCA = obeticholic acid; CP = cyclophosphamide; PlGF = placental growth factor; NF-κB = nuclear factor-kappa B; TNF-α = tumor necrosis factor-alpha; SIRT1 = Sirtuin type 1; Nrf2: nuclear factor erythroid 2-related factor 2; Myd88 = myeloid differentiation factor 88; TLR4 = toll like receptor 4; FOXO1 = Forkhead-box transcription factor1

The placenta serves as a conduit between the fetus and the mother. Normal maternal-fetal circulation is essential for the fetus nutrition and oxygen delivery, waste elimination, and defense against xenobiotics. Preterm birth, neonatal cerebral palsy, and fetal growth limitation are among the pregnancy issues caused by toxicity and, consequently, the placenta’s inability to nourish the fetus. CP is one of the chemotherapeutic medicines used to treat autoimmune disorders and cancers [5]. Remarkably, exposures to CP throughout the second and third trimesters have been documented in cases when severe maternal conditions such rheumatoid arthritis and SLE necessitates treatment during pregnancy [21]. In the present investigation, CP caused placental damage.

OCA, a semisynthetic derivative of chenodeoxycholic acid, is a potent and selective FXR agonist that is used to treat biliary cholangitis in patients with compensated cirrhosis or without. OCA exhibits antioxidant and anti-inflammatory qualities in a number of models [16].

In the present study, CP significantly decreased placental wt which is in line with Abdelzaher and her colleges [5] which reported that CP associated with significant placental wt reduction and histological alterations of the placenta. On the other hand, PlGF; a member of the family of vascular endothelial growth factors, was linked to a decrease in placental weight. PlGF is mostly expressed in the placenta, but it is also faintly expressed in the skeletal muscle, heart muscle, liver, lungs, and bone. PlGF is pro-angiogenic, meaning it encourages the growth and maturation of the placental vascular system. Additionally, women who develop pre-eclampsia have been found to have low PlGF concentrations. Therefore, placental damage may be linked to the decrease in PlGF with CP as low PlGF is a sign of abnormal placentation [22].

When OCA was given with CP, placental wt increased with improvement in histological picture. It also, dramatically boosted PlGF as compared to the CP group. This was consistent with earlier studies that found the same effect in different models of placental injury and attributed it to OCA anti-oxidant and anti-inflammatory protective properties [13].

Running in the same stream, CP induced placental oxidative stress as evidenced by increment in MDA together with reduction of TAC. Our results could be explained by earlier research showing that CP increases intracellular reactive oxygen species (ROS) generation, which can result in oxidative stress-induced biochemical and physiological abnormalities. TAC’s inactivation to one or more sulfhydryl group residues in the antioxidant enzymes that are responsible to their catalytic activity caused CP to decrease level of TAC [23–25].

A considerable proportion of Nrf2 is used by the release of ROS. By triggering the Nrf2 signaling pathway, OCA improves detoxification and the antioxidant defense system. The current work indicates that OCA helps the protein withstand oxidative stress by raising Nrf2 levels and preventing CP-induced Nrf2 depletion. Nrf2 also plays a role in the inflammatory process as a major negative regulator of the release of inflammatory cytokines [26, 27]. On the other hand, SIRTI can boost and activate Nrf2’s transcriptional activity. Conversely, SIRT1 downregulation significantly reduced Nrf2 protein expression. The SIRT1/Nrf2 pathway can prevent oxidative stress-induced placental damage by increasing the body’s antioxidant capacity [28].

SIRT1 amplifies the cell cycle arrest that FOXO induces by increasing its deacetylation. Following FOXO deacetylation, ubiquitination can break down FOXO, reducing its concentration and preventing cell death, so shielding cells from oxidative stress [29]. According to Salama and her colleagues [30], controlling the SIRT1/FOXO1 pathway is essential for treating inflammatory lung conditions and avoiding tissue damage in rats. Importantly, the degree of FOXO deacetylation regulates the complex and dynamic processes of cell death and the cell cycle, as well as oxidative stress. Thus, studying the role of the SIRT1/FOXO1 pathway in toxic damage is essential [28]. To further elucidate the mechanism of protection that OCA is believed to produce, measures of the SIRT1/Nrf2 and SIRT1/FOXO1 signaling pathways in placental tissue were examined. Current research indicates that CP suppresses SIRT1 and increase FOXO1 immunohistochemical expression, which OCA activates SIRT1 with negatively immunohistochemical expression of FOXO1.

The results of the present study demonstrated that CP markedly elevated the placental Myd88 level, TLR4 immunohistochemistry expression, and gene expression of both NF-κB and TNF-α. Since TLR is a key regulator of inflammation, activating TLR4 through Myd88 and then stimulating NF-κB translocation into the nucleus increased the amounts of many pro-inflammatory factors, including TNF-α and IL-1β. In various animal models, such as ischemia/reperfusion, Alzheimer’s disease, and autoimmune illnesses, TLR4 controls inflammation and tissue damage [31].

When compared to the CP group, the co-administration of OCA markedly reduced the inflammatory indices. OCA and its derivatives have been demonstrated in earlier research to reduce the release of proinflammatory cytokines in acute liver failure, osteoarthritis and cardiac toxicity [16, 32, 33]. By suppressing TLR4 expression, OCA reduced the inflammatory response by negatively regulating the Myd88-dependent TLR4/NF-κB signal transduction, which in turn lowered the transcription of NF-κB. OCA decreased NF-κB activity via the TLR4 Myd88-dependent pathway.

Since Sirt1’s N-terminal domain encourages caspase-3 deacetylation, it directly inhibits caspase-3 activation. Furthermore, it was shown that Sirt1 reduced the generation of ROS in renal mesangial cells caused by advanced glycation end products by activating the Nrf2 pathway [34]. All of them show that Sirt1 has a crucial role in controlling apoptosis, inflammation, and antioxidant defense systems, helping to prevent CP-induced placental damage by OCA.

Briefly, a reciprocal relationship exists, where SIRT1 dependant signaling pathways exerts anti-inflammatory and antioxidant effects, while TLR4/MyD88/NF-κB activation promotes pro-inflammatory responses. The balance between these pathways likely dictates the extent of placental injury and recovery [6, 7].

The findings of this study have to be seen in light of some limitations; Western blot for NF-κB and TNF-α would have strengthened the claim, in further study Western will be done.

## Conclusion

The current study introduces OCA as a novel therapeutic approach that reduces CP-induced placental injury via OCA regulation to SIRT1/Nrf2/TNF-α; SIRT1/FOXO1 and TLR4/Myd88 /NF-κB signaling pathways in conjunction with its antioxidant, anti-inflammatory, and anti-apoptotic properties.

## Data Availability

No datasets were generated or analysed during the current study.

## Abbreviations

CP: Cyclophosphamide
FOXO1: Forkhead-box transcription factor1
FXR: Farnesoid X receptor
IL-1β: Interleukin-1β
Myd88: Myeloid differentiation factor 88
NF-κB: nuclear factor-kappa B
Nrf2: Nuclear factor (erythroid-derived 2)-like 2
OCA: Obeticholic acid
PlGF: Placental growth factor
SIRT1: Sirtuin type 1
SLE: Systemic lupus erythematosus
TLR4: Toll- like receptor4
TNF-α: Tumor necrosis factor-α
